# Novel Development and Sensory Evaluation of Extruded Snacks from Unripe Banana (Musa ABB cv. Kluai ‘Namwa’) and Rice Flour Enriched with Antioxidant-Rich *Curcuma longa* Microcapsules

**DOI:** 10.3390/foods14020205

**Published:** 2025-01-10

**Authors:** Nurulhusna Awaeloh, Surasak Limsuwan, Pinanong Na-Phatthalung, Thammarat Kaewmanee, Sasitorn Chusri

**Affiliations:** 1 Biomedical Technology Research Group for Vulnerable Populations and School of Health Science, Mae Fah Luang University, Muang, Chiang Rai 57100, Thailand; 6451811004@lamduan.mfu.ac.th; 2 Traditional Thai Medical Research and Innovation Center, Faculty of Traditional Thai Medicine, Prince of Songkla University, Hat Yai 90110, Thailand; surasak.l@psu.ac.th; 3 Division of Hematology and Oncology, Icahn School of Medicine at Mount Sinai, New York, NY 10029, USA; pinanong.naphatthalung@mssm.edu; 4 Department of Food Science and Nutrition, Faculty of Science and Technology, Prince of Songkla University, Muang, Pattani 94000, Thailand

**Keywords:** *Curcuma longa*, herbal-derived antioxidants, functional food, herbal-based functional ingredient, extruded snack, unripe banana flour, gluten-free flour

## Abstract

With the growing consumer demand for natural functional ingredients that promote health and well-being while preventing age-related diseases, this study aimed to develop extruded snacks enriched with *Curcuma longa* (turmeric) microcapsules, recognized for their significant antioxidant properties. Unripe banana flour (Musa ABB cv. Kluai ‘Namwa’) and rice (*Oryza sativa*) flour were employed as a gluten-free base to create this novel extruded snack. *Curcuma longa* extract microcapsules were prepared using a spray-drying technique with varying core-to-wall ratios. Antioxidant capacities were assessed through DPPH, ABTS, superoxide radical scavenging, metal chelating, and ferric-reducing assays. The CM6 microcapsules, prepared at 140 °C with a 1:10 core-to-wall ratio, exhibited potent antioxidant activity, with 58.93 ± 3.31% inhibition for DPPH radicals, 87.58 ± 1.33% for ABTS, and 78.41 ± 1.40% for superoxide radicals. Snacks enriched with 0.25% CM6 microcapsules received high consumer acceptance, with an average liking score of 7.5 out of 9. These findings suggest that snacks made with these gluten-free flours and *Curcuma longa* microcapsules could be novel, convenient, and appealing functional food products that offer an attractive way to deliver antioxidant benefits with high consumer acceptance. Further research on evaluating the active constituents in the snack, its long-term health benefits, and shelf-life stability is recommended for commercialization.

## 1. Introduction

Due to their vast array of health benefits, the growing interest in plant-based bioactive compounds has led consumers and researchers to explore their potential as functional ingredients in food products in recent years [1,2,3]. These bioactive compounds, particularly antioxidant-rich compounds, play a significant role in mitigating the effects of oxidative stress, which is implicated in the progression of several metabolic and neurodegenerative diseases, including cardiovascular diseases and Alzheimer’s disease [1,2,3]. Furthermore, both preclinical and clinical studies have demonstrated that consuming food supplemented with functional ingredients, such as fermented milk enriched with pomegranate peel extract [4], yogurt fortified with olive polyphenol [5], bread enriched with phytosterols and curcumin [6], and macaroni fortified with green tea and turmeric curcumin extract [7], enhances the activities of antioxidant enzymes, decreases oxidative stress, and lowers elevated cholesterol and triglyceride levels. Therefore, developing novel functional-enhanced food products containing plant-based bioactive substances with health-promoting effects is a promising approach. In particular, the increasing demand for foods rich in antioxidants, which can prevent oxidative stress-mediated diseases, aligns with the steady rise in the global aging population.

Curcumin, a yellow polyphenolic pigment isolated from the rhizome of *Curcuma longa*, possesses remarkable health benefits and exhibits preventive mechanisms against the progression of several non-communicable chronic diseases [8,9]. Although it is commonly employed in food products mainly as a coloring agent, its use as a functional ingredient in the food industry is limited. Its poor bioavailability due to poor absorption, rapid liver and intestinal wall metabolism, and rapid systemic elimination have been shown to limit its use [10,11]. Additionally, a recent study demonstrated that formulating probiotic yogurt with 0.2% (*w*/*w*) of standard *Curcuma longa* extract altered the yogurt’s flavor, introducing a bitter taste [12]. Various strategies, such as combining with another substance [13] or utilizing encapsulation technology [14,15,16,17], have been developed for this plant to counter these drawbacks, enhance curcumin’s bioavailability, and mask its unpleasant taste.

The extrusion process, widely utilized in the food industry for the preparation of various snacks, was employed to demonstrate the feasibility of incorporating curcumin-containing microcapsules into extruded snacks made from green banana flour and rice flour. The numerous benefits of the extrusion process and the resulting extruded snacks have been well documented [18,19]. This process enhances the nutritional quality of snacks by allowing precise control over time and temperature, thereby minimizing nutrient losses [19]. Extrusion also results in the gelatinization of starches and the breakdown of complex proteins, making these macronutrients more accessible and improving digestibility [20,21]. Additionally, extrusion is an energy-efficient process that contributes to improved shelf life and enhanced sensory qualities of the products [18,19]. Green banana flour and rice flour were chosen for their health benefits, such as high antioxidant properties, significant amounts of resistant starch, and their suitability as safe and effective alternatives for individuals with gluten intolerance or celiac disease [22,23].

This study aims to assess the possibility of utilizing microcapsules containing standardized *Curcuma longa* extract in extruded snacks and evaluate the consumer acceptability of this novel product. These microcapsules, which were prepared using a spray-drying technique, were employed to improve the sensory properties of curcumin and enrich extruded snacks made from two types of gluten-free flour: unripe banana flour (Musa ABB cv. Kluai ‘Namwa’) and rice (*Oryza sativa*) flour. By enhancing the antioxidant properties of these snacks without compromising taste or texture, this research seeks to offer a novel functional food product that caters to the growing consumer demand for antioxidant-enriched foods.

## 2. Materials and Methods

### 2.1. The Standardized Extract of Curcuma longa and Chemicals

The standardized water extract of *Curcuma longa* rhizome, with a curcumin content of 5% (*w*/*w*), was purchased from the AP Operations Co., Ltd., Chonburi, Thailand (Code No.: EXP-TUM-3A). The extract appears as a light-yellow fine powder with moisture and ash contents of less than 5% (*w*/*w*). The extract powder was passed through an 80-mesh (236 µm) sieve, packed in a tight-seal aluminum-laminated bag, and stored at 4 °C until further use. Food-grade maltodextrin with a dextrose equivalence (D.E.) of 10–15 and gum Arabic were purchased from Scienfield Expertise PLT. (Shah Alam, Selangor, Malaysia). To evaluate the antioxidant activity and determine phenolic compounds and flavonoids, the extract and its microcapsules were prepared by dissolving them in 95% ethanol to obtain the desired concentration unless stated otherwise.

### 2.2. Encapsulation of the Standardized Extract of Curcuma longa

The coating materials, maltodextrin and gum Arabic, were dissolved completely in distilled water at a 1:5 (*w*/*w*) ratio overnight on a magnetic stirrer. The ratio was 8:2 for CM1 and CM4 and 6:4 for CM2, CM5, CM3, and CM6. The following spray-drying process parameters used in this present work were obtained from our previous study [24]. The feeding solutions were prepared by adding the extract at a concentration of 10 mg/mL in 95% food-grade ethanol into the coating material solution at a ratio of 1:20 for CM1-3 and 1:10 for CM4-6 (*v*/*v*; core material to coating material) using vigorous magnetic stirring. Before being subjected to a laboratory-scale spray dryer (Buchi Mini Spray Dryer B-290, Fawil, Switzerland), the feeding solutions with different ratios of the core to coating material were separately homogenized at 13,500 rpm for 3 min (Ultra-Turrax, Staufen, Germany). The drying procedure, with the aspirator rate set to 100% throughout the experiment, was operated under the following conditions: The inlet and outlet temperatures were set at 140 ± 1 °C and 80 ± 2 °C, respectively. A feed flow rate of 10 mL/min was maintained [24]. The obtained *Curcuma longa* microcapsules (CMs), harvested from the collecting vessel below the drying cyclone chamber, were stored at 4 °C in a UV-protected airtight container until use.

### 2.3. Estimation of Total Phenolic and Total Flavonoid Contents of CMs 

The total phenolic content (TPC) of the *Curcuma longa* microcapsules (CMs) was estimated based on the Folin–Ciocalteu procedure with slight modifications [25]. Briefly, 125 µL of the Folin–Ciocalteu reagent was added to a 96-well plate containing an aliquot of the extract (15 µL) at a concentration of 2.5 mg/mL, prepared by dissolving the microcapsule extract in 95% ethanol, and mixed well. Sodium carbonate (125 µL; 20% *w*/*v*) was then added to each well. The mixtures were incubated in the dark at 37 °C for 1 h, and their optical densities at 725 nm were measured using a microplate reader (Spark^®^ Multimode Microplate Reader, Männedorf, Switzerland). The TPC was calculated using a gallic acid calibration curve and expressed as mg gallic acid equivalent per g of extract (mg GAE/g extract).

The total flavonoid content (TFC) of the samples was measured using the aluminum chloride colorimetric assay with minor modifications [25]. An aliquot of the extract (2.5 mg/mL) was thoroughly mixed with 6 µL of 5% (*v*/*v*) NaNO_2_ and 6 µL of 10% AlCl_3_ in a 96-well plate. The plate was then kept in the dark at 37 °C for six minutes, followed by adding 40 µL of 1 M NaOH. The absorbance of each well was recorded at 510 nm using a microplate reader (Spark^®^ Multimode Microplate Reader, Männedorf, Switzerland). The TFC per gram of extract was calculated based on a catechin calibration curve and presented as mg catechin equivalent per g of extract (mg CAE/g extract).

### 2.4. Determination of Antioxidant Capacities of CMs 

The metal chelating activity (MCA) was used to express the Fe^2+^-binding abilities of the samples [26]. The CMs (2.5 mg/mL; 125 µL) were thoroughly mixed with an aliquot of 2 mM FeCl_2_ (12.5 µL), and the levels of Fe^2+^–ferrozine formation were monitored at 562 nm after a 10 min incubation at room temperature using a microplate reader (Spark^®^ Multimode Microplate Reader, Männedorf, Switzerland). The standard chelator, EDTA, was tested in parallel as a positive control. The percentage chelating ability of the samples and standards was determined using the following equation:

% chelating ability = [(OD of Control−OD of Sample)/OD of Control] × 100.

The free radical scavenging properties of the CMs were tested against 2,2′-azino-bis-3-ethylbenzthiazoline-6-sulfonic acid (ABTS) and 1,1-diphenyl-2-picrylhydrazyl (DPPH) radicals, according to previously established protocols with some modifications, as mentioned below [25,27]. The samples were serially 2-fold-diluted with dimethyl sulfoxide (DMSO; 50% *v*/*v*) to achieve concentrations ranging from 1.22 to 2500 µg/mL. Trolox at concentrations of 0.61 to 1250 µg/mL was tested in parallel as an antioxidant control. The optical density of the solution at 517 nm was measured using a microplate reader (Spark^®^ Multimode Microplate Reader, Männedorf, Switzerland), and the radical scavenging activity was reported as the percentage inhibition of DPPH or ABTS free radicals by CMs using the following formula:

% DPPH/ABTS radical scavenging activity = [(OD of Control−OD of Sample)/OD of Control] × 100.

The reducing power of CMs was determined using a ferric-reducing antioxidant power (FRAP) assay [25,27]. The extract (2.5 mg/mL; 30 µL) was added to a 96-well plate containing 270 µL of FRAP reagent. The levels of Fe^2+^–tripyridyltriazine (blue-colored complex) were recorded at 593 nm using a microplate reader (Spark^®^ Multimode Microplate Reader, Männedorf, Switzerland), as a result of the reduction of the Fe^3+^–TPTZ complex (colorless complex) by an electron-donating antioxidant and reported as FRAP values, which were calculated from the calibration curve of a freshly prepared working solution of FeSO_4_.

To evaluate the scavenging properties of CMs towards superoxide anion, the nitroblue tetrazolium (NBT) assay was employed. As described previously [27], the riboflavin/methionine/illuminate system was used to generate the superoxide radical. The results were recorded based on the reduction of NBT to form a purple-colored formazan (NBT^2+^) caused by the generated superoxide radicals. The absorbance of the formazan dye was measured at 560 nm against an appropriate blank solution using a microplate reader (Spark^®^ Multimode Microplate Reader, Switzerland). Catechin was included as a positive control. The capability to scavenge superoxide radicals was calculated using the following formula:

% superoxide radical scavenging activity = [(OD of Control−OD of Sample)/OD of Control] × 100.

### 2.5. Preparation of Extruded Snack

Rice flour (Sang Yod Phattalung) was dry-milled into a fine powder and stored at 30 ± 2 °C for no more than three days before use. Unripe banana flour was purchased from Musarium Co., Ltd., and pregelatinized using drum drying at 120 ± 1 °C. Simultaneously, this flour was further milled into a fine powder. The feed blend for the extrusion consisted of 76.5 g/100 g rice flour and 20 g/100 g pregelatinized unripe banana flour. Before extrusion, the paste was wrapped in aluminum foil bags and held at 25 ± 1 °C.

The extrusion experiment was carried out on a co-rotating twin-screw extruder (Hermann Berstorff Laboratory Co-rotating Twin Screw Extruder, Hannover, Germany), which comprised a barrel with six sections and a barrel length-to-diameter ratio (L/D) of 870:25. A single-screw feeder was used to feed the raw materials into the extruder with a feeding valve of 20 L per hour and a feeding rate of 20 kg/h. The barrel temperature profile was set as follows: 40 °C (Section 1), 80°C (Section 2), 110 °C (Section 3), 120 °C (Section 4), 144°C (Section 5), and 140 °C (Section 6). The temperature of the 24.5 mm thick die plate with one slit (1 mm × 20 mm) was 140–150 °C. The screw speed was set at 400 rpm. The extrudates were cut at 600 rpm using a speed die face cutter with four-bladed knives. The extruded snacks were dried for 10 min in a forced-air oven at 80 °C. The samples were collected, packed in vacuum-wrapped aluminum foil bags, and stored at 25 °C until further use.

### 2.6. Analysis of Nutritional Facts

Bags of the extruded snacks were randomly taken to analyze their nutritional fact values at the Institute of Food Research and Product Development, Kasetsart University, Bangkok, Thailand. The energy value per 100 g was calculated based on the Thai Recommended Daily Intake (Thai RDI) and the guidelines set by the Thai FDA, using the following conversion factors: carbohydrate—4 kcal/g; protein—4 kcal/g; and fat—9 kcal/g. The analysis items, including total energy, energy from fat, and total carbohydrate, were measured according to the established methods of the International Officers and Committees 1993 with slight modifications [28,29]. Other contents, including total fat, saturated fat, cholesterol, dietary fiber, sugar, sodium, vitamins B1 and B2, calcium, iron, and moisture, were measured according to the methods prescribed by the Official Methods of Analysis, the 21st Edition, AOAC International, Rockville, MD, USA, with slight modifications [29,30]. The contents of vitamin A and ash per 100 g of samples were measured based on previously described protocols [31].

### 2.7. Sensory Evaluation

The sensory evaluation was conducted using the consumer technology analysis program provided by Zenme Co., Ltd. (Chiang Mai, Thailand). The test included 100 volunteers from various regions in Thailand, categorized into four different age groups (20–29, 30–39, 40–49, and >50 years). More than 90% of the panelists were interested in testing and purchasing the extruded snack. The consumers were classified into a healthy and regular behavior group based on their exercise, food consumption, drinking, and sleeping duration. Before testing, 10 g of the snack was sealed in a pouch. Panelists were required to evaluate the appearance, odor, crispness, taste, and overall acceptability of the extruded snacks using a 9-point hedonic scale (1 = dislike extremely, 9 = like extremely).

### 2.8. Statistical Analysis

All experiments were performed in triplicate, and the results are expressed as the mean ± SD. The means of all parameters were assessed for statistical significance utilizing one-way ANOVA followed by Duncan’s New Multiple Range Test. Statistically significant values were considered at *p* < 0.05. Data were analyzed using SPSS for Windows Version 19 (IBM Corp. Released 2010, IBM SPSS Statistics for Windows, and Version 19.0. Armonk, NY: IBM Corp.).

## 3. Results

The six types of microcapsule powders, CM1-CM6, were produced with a standardized extract of *Curcuma longa* as the core material, and the wall material was either maltodextrin (MD) or gum Arabic (GA). The microcapsules CM1 and CM4 were made using MD as the wall material with a core-per-wall ratio of 1:20 and 1:10, respectively. The combination of MD with GA, at a ratio of either 8:2 or 6:4, was used as the wall material in the preparation of CM2, CM3, CM5, and CM6, with a core-per-wall ratio of 1:20 for CM2 and CM3 and 1:10 for CM5 and CM6. As demonstrated in Table 1, the microcapsules obtained with a core-to-wall ratio of 1:10 exhibited the highest phenolic and high flavonoid content, particularly CM4 and CM6. Curcumin-based microcapsules that used only MD as a wall material exhibited slightly lower phenolic and flavonoid content.

Furthermore, their antioxidant properties were assessed using various methods, including their reducing and metal chelating abilities and their radical scavenging activities against DPPH, ABTS, and superoxide radicals. As described in Table 2, all microcapsules exhibited similar percentages of metal chelating inhibition, ranging from 50 to 65%, except for CM4 and CM5, which had slightly lower activities. The reducing ability, expressed by the FRAP value, was found to be in the range of 16–28 µM FeSO_4_/mg of the sample, with CM4 exhibiting the highest reducing capability at 28.95 ± 1.47 µM FeSO_4_/mg. The percentages of free radical scavenging effects against DPPH, ABTS, and superoxide radicals were found to be in the ranges of 47–70%, 62–100%, and 50–93%, respectively. Notably, CM6 exhibited slightly better free radical scavenging properties, with inhibition percentages of 58.93 ± 3.31 for DPPH radicals, 87.58 ± 1.33 for ABTS radicals, and 78.41 ± 1.40 for superoxide radicals. Therefore, this microcapsule was chosen for use in the preparation of the extruded snack.

**Figure 1:**
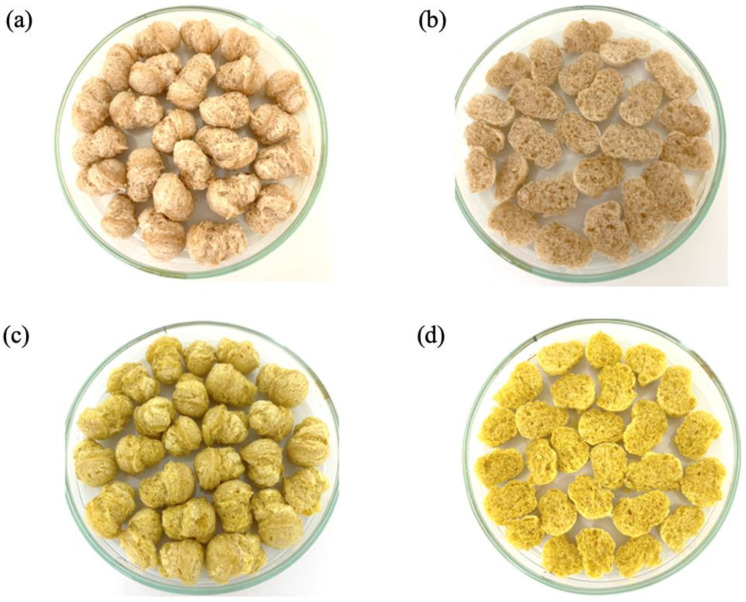
Appearance of functional food extruded snacks: external appearance of control formula (**a**), internal appearance of control formula (**b**), external appearance of curcumin microcapsule (**c**), and internal appearance of curcumin microcapsule (**d**).

The primary ingredients used in preparing the novel extruded snack were local rice flour and pregelatinized unripe banana flour combined with 0.25% CM6 (Table 3). Figure 1 illustrates the external and internal appearances of both the novel and control snacks without curcumin microcapsules, highlighting their texture and color. A comparison between the control and novel formulas revealed no visible texture changes. However, the snack containing CM6 microcapsules was noticeably more yellow in color intensity.

The energy value of this snack was 366.20 kcal/100 g, with no energy derived from fat. The novel food contained 84.41 g of carbohydrates, 7.14 g of protein, 216.06 mg of sodium, 133.35 mg of calcium, and 2.57 mg of iron per 100 g of snack. Additionally, the tested snack included 2.50 g of fiber, 1.48 g of ash, and 6.97 g of moisture per 100 g.

To preliminarily assess the consumer acceptability of the developed snack, sensory evaluation data were collected from volunteers exhibiting either healthy food consumption behaviors (HFCbs; *n* = 50) or regular food consumption behaviors (RFCbs; *n* = 50). The majority of volunteers from both groups were aged between 20 and 49 and earned between THB 15,000 and 45,000 per month. Volunteers in the HFCb group typically consumed healthy foods, did not have drinking habits, exercised more than three times a week, and regularly attended health checkups. Except for the crispiness of the product, the scores for sensory characteristics obtained from the HFCb volunteers were significantly higher than those from the RFCb volunteers. Both groups rated the crispiness similarly, with no significant difference (7.4 ±1.2 for HFCb and 6.8 ±1.7 for RFCb). The HFCb group rated its overall acceptability, appearance, odor, taste, and texture higher (7.2–8.0) than the RFCb group (6.5–7.5) (Table 4).

## 4. Discussion

The findings of this study suggest that the microencapsulation of *Curcuma longa* extract, particularly in the form of CM6 microcapsules, can significantly enhance the antioxidant properties of extruded snacks made from unripe banana flour. The CM6 microcapsules demonstrated strong antioxidant capacities, as evidenced by various assays, including DPPH, ABTS, and FRAP. These results align with previous studies highlighting curcumin’s potential as a potent antioxidant in functional food products. However, the present study demonstrated this further by successfully incorporating these microcapsules into an extruded snack format, which has broader consumer appeal and practical applications in the food industry.

The extrusion technique was chosen for snack preparation due to its low operational costs, flexibility, high productivity, and significant nutrient retention. Additionally, extruded foods are microbiologically safe, possess lower moisture content, and have a longer shelf life [17,18]. Given the high prevalence of gluten-related disorders [32], two types of naturally gluten-free flour—local rice flour and unripe banana flour—were used in incorporating CM6 into the food system [33,34]. Although the supplementation of plant-derived antioxidants has been reported to enhance the consumer acceptability, biological activities, and storage stabilities of various foods [34,35,36,37,38], the development of beverages and food enriched with curcumin has resulted in unpleasant tastes and odors [7,39,40]. Our results showed that extruded snacks made from flour fortified with CM6 achieved high consumer acceptability (7.2–8.0), comparable to ice cream and yogurt supplemented with microcapsules of pomegranate peel phenolics [41,42] and chocolate bars enriched with microcapsules containing polyphenols from cocoa bean shells [43], which received liking scores of approximately 7–8.5. Additionally, incorporating encapsulated phenolics isolated from sour cherries into cakes could mask the unpleasant bitter taste and improve the cakes’ color attributes and acceptability [44]. This is consistent with our findings, where the CM6 microcapsules maintained their antioxidant properties without significantly altering the sensory profile of the extruded snacks.

Various studies have highlighted challenges related to incorporating curcuminoids, curcumin, or standardized extracts of *Curcuma longa* into the food industry, including a lack of long-term stability, poor water solubility, bitterness, and astringency [7,10,11,15]. Encapsulation technologies have emerged as crucial in the functional food sector in addressing these limitations [15,16,17]. In the current study, we optimized the spray-drying process for preparing the standardized *Curcuma longa* extract microcapsules using a mixture of maltodextrin and gum Arabic in a 6:4 ratio as wall materials. The optimal core-to-wall ratio was determined to be 1:10 with an inlet temperature of 140 °C (CM6). The spray-drying technique was selected for microcapsule preparation owing to its cost-effectiveness, energy-efficient operation, high encapsulation efficiency, and the ensured stability of the encapsulated products [45].

These microcapsules exhibited significant antioxidant properties through either preventive effects or chain-breaking mechanisms. Curcumin, the well-known active principle in the standardized extract of *Curcuma longa*, has been extensively described as a potent natural chelator that plays a crucial role in preventing the generation of reactive oxygen species (ROS) through Haber–Weiss reduction followed by Fenton reactions due to excess free iron [46,47]. The α,β-unsaturated β-diketo moiety of curcumin effectively binds to active redox metals such as Cu^2+^, Fe^2+^, Mn^2+^, Pb^3+^, etc., through chelation. This chelating ability has been shown to prevent amyloid aggregation—a critical factor in Alzheimer’s pathogenesis [48]—and to suppress tumorigenic actions caused by iron overload in epithelial cell lines (T51B cells) and liver epithelial-like cells (RL-34 cells) [49].

Furthermore, the CM6 microcapsules also demonstrated chain-breaking properties similar to curcumin. Alongside scavenging activity toward DPPH, ABTS, and superoxide radicals observed in CM6 and previously reported for curcumin and *Curcuma longa* extracts [50,51], the scavenging effects of this plant extract and its isolated compounds have also been proven against hydrogen peroxide, hydroxyl radical, singlet oxygen, and nitric oxide [52]. These radical scavenging effects are recognized as one of the primary mechanisms in preventing the progression of various diseases, including cancers [53], cardiovascular diseases [54], and neurodegenerative disorders [55].

While these findings are promising, this study has limitations. As curcumin is known to degrade over time, the long-term stability of the microencapsulated curcumin and the antioxidant properties of the developed snack during storage should be considered in future studies. Additionally, while this study focused on antioxidant activity, other potential health benefits of curcumin, such as preventive effects towards low-grade chronic inflammation or oxidative stress-induced damage of biomolecules and glycation, were not explored. Conducting in vivo and clinical studies to assess the health benefits of consuming these snacks would be crucial in establishing their value as functional foods.

Furthermore, the consumer acceptability results from the sensory evaluation indicate that the developed extruded snacks were well received, particularly in appearance, taste, and overall acceptability. However, certain sensory characteristics, such as crispiness, remain to be optimized to appeal to a broader consumer base. The differences observed between the healthy and regular food consumption groups suggest that targeted marketing strategies might be necessary to position this product effectively in the marketplace.

In conclusion, this study has successfully developed a novel functional snack by incorporating antioxidant-rich *Curcuma longa* microcapsules into unripe banana flour. However, further studies are urgently required to confirm their health benefits for consumers and their shelf life, storage stability, and market feasibility. By addressing these areas, this study could pave the way for developing new functional foods to prevent oxidative stress-related diseases, particularly in aging populations.

## 5. Conclusions

This study has successfully demonstrated the potential of using *Curcuma longa* microcapsules, developed through a spray-drying technique, to enrich extruded snacks made from unripe banana flour. The *Curcuma longa* microcapsule CM6 showed significant antioxidant activity, as measured by multiple assays, including DPPH, ABTS, and FRAP, without compromising the snacks’ sensory qualities. The results from the sensory evaluation indicate that the novel snacks were well received, especially among health-conscious consumers, with high ratings for taste, appearance, and overall acceptability. The findings highlight the potential of integrating antioxidant-rich microcapsules into gluten-free flour products. This represents an innovative approach to developing functional foods that cater to modern consumer demands for health-promoting, convenient snacks. Despite these promising findings, further research is needed to explore the long-term health benefits of consuming these snacks. In vivo models and clinical studies evaluating their impact on oxidative stress markers and other health-related outcomes would provide valuable insights. Additionally, assessing the shelf-life stability of the snacks will be crucial for determining their market potential.

## Figures and Tables

**Table 1:** Total phenolic content and total flavonoid content of different microcapsules containing the standardized extract of *Curcuma longa*.

Microcapsules	Total Phenolic Content		Total Flavonoid Content
(mg gallic acid equivalence/g of extract)		(mg catechin equivalence/g of extract)	
CM1	278.17 ± 26.20 ^b^		61.13 ± 2.78 ^c^
CM2	289.79 ± 26.58 ^b^		122.64 ± 5.95 ^a^
CM3	282.04 ± 19.46 ^b^		82.59 ± 5.93 ^b^
CM4	366.90 ± 30.32 ^a^		82.03 ± 2.47 ^b^
CM5	270.80 ± 18.93 ^b^		89.08 ± 4.23 ^b^
CM6	346.36 ± 24.00 ^a^		91.14 ± 5.96 ^b^

^a–c^: values in the same row with different superscripts are significantly different (*p* < 0.05).

**Table 2:** Antioxidant capacities of different microcapsules containing the standardized extract of *Curcuma longa*.

Microcapsules	Mean ± SD *				
	Metal Chelating	DPPH	ABTS	FRAP	NBT
CM1	61.52 ± 0.87 ^a^	47.39 ± 4.15 ^c^	58.42 ± 1.52 ^d^	16.85 ± 0.10 ^d^	50.50 ± 4.90 ^c^
CM2	61.42 ± 1.28 ^a^	70.72 ± 3.39 ^a^	105.22 ± 1.73 ^a^	17.25 ± 0.65 ^d^	93.84 ± 1.55 ^a^
CM3	64.65 ± 1.75 ^a^	47.36 ± 1.25 ^c^	62.28 ± 2.30 ^d^	19.37 ± 1.93 ^c^	93.24 ± 3.76 ^a^
CM4	52.41 ± 4.28 ^b^	58.90 ± 0.46 ^b^	78.73 ± 3.27 ^c^	28.95 ± 1.47 ^a^	84.69 ± 8.02 ^b^
CM5	50.57 ± 1.99 ^b^	59.86 ± 1.12 ^b^	81.72 ± 3.24 ^c^	18.93 ± 0.49 ^c,d^	85.32 ± 2.01 ^b^
CM6	62.66 ± 3.29 ^a^	59.89 ± 3.31 ^b^	87.58 ± 1.33 ^b^	22.88 ± 1.05 ^b^	78.41 ± 1.40 ^b^

* For the DPPH radical scavenging activity assay, ABTS radical scavenging activity assay, and nitroblue tetrazolium (NBT) assay, the results are expressed as the mean percentage of inhibition ± SD, while the results obtained from the ferric-reducing antioxidant power (FRAP) assay are presented as µM FeSO_4_/µg of extract. ^a–d^: values in the same row with different superscripts significantly differ (*p* < 0.05).

**Table 3:** Blend preparation of the novel extruded snack.

Ingredients (in 100 g)	Control Formula	Curcumin Microcapsule Formula
Local rice flour (Sang Yod Phattalung)	76.5	76.5
Green banana flour	20	20
Salt	0.75	0.75
Rice bran oil	1	1
Stabilizer (INS460ii): cellulose	1	1
Sodium carbonate	0.5	0.5
CM-6 powder/wall material powder	0.25	0.25

**Table 4:** Sensory data for an extruded snack made from Cplus in volunteers with healthy food consumption behaviors (HFCbs) and regular food consumption behaviors (RFCbs).

Characters	Groups of Volunteers	
	HFCb (n = 50)	RFCb (n = 50)
Overall acceptability	7.8 ± 1.1 ^a^	6.9 ± 1.5 ^b^
Appearance	7.5 ± 1.2 ^a^	6.7 ± 1.5 ^b^
Odor	7.2 ± 1.4 ^a^	6.5 ± 1.5 ^b^
Crispiness	7.4 ± 1.2 ^ns^	6.8 ± 1.7 ^ns^
Taste	7.8 ± 1.2 ^a^	6.9 ± 1.7 ^b^
Texture	8.0 ± 1.0 ^a^	7.5 ± 1.4 ^b^

^a,b^: values in the same row with different superscripts are significantly different (*p* < 0.05). ^ns^: values are not significantly different (*p* < 0.05).

## Data Availability

The original contributions presented in the study are included in the article, further inquiries can be directed to the corresponding author.
